# Dry-powder inhalers in patients with persistent airflow limitation: usability and preference

**DOI:** 10.1186/s40248-016-0068-x

**Published:** 2016-09-05

**Authors:** Roberto W. Dal Negro, Massimiliano Povero

**Affiliations:** 1 National Centre for Respiratory Pharmacoeconomics & Pharmacoepidemiology - CESFAR, Verona, Italy; 2 AdRes Health Economics and Outcome Recourses, Torino, Italy

**Keywords:** Breezhaler, Bronchial Asthma, COPD, Genuair, Handihaler, Handling Questionnaire, Patients' preference, Patients' usability

## Abstract

**Background:**

Inhalation devices represent *per sé* critical factors because they can affect the therapeutic outcomes independently of the drug used. The role of patients’ usability and preference (PUP) for Dry Powder Inhalers (DPIs) is high indeed because they can influence the extent of the adherence to treatment and the therapeutic outcomes.

Aim of the study was to assess and compare the PUP of three different DPIs in out-patients with persistent airflow limitation due to asthma or COPD.

**Methods:**

The PUP of three different DPIs (Breezhaler; Genuair; Handihaler) were investigated by means of the *Handling Questionnaire* in out-patients with persistent airflow limitation needing an inhalation therapy*.* Patients had to report their preference before and after the nurse’s instruction on the handling of each device. The nurse had also to note the critical steps during the patient’s procedure for actuation; to count the number of attempts needed for actuating the device properly, and to measure the time (in sec.) required for these procedures. Data were collected up to three attempts per device.

Statistics: Welch test was used for normal distributed variables, while the Wilcoxon test for not normal distributed variables. The *χ*^2^ test and the ANOVA test were also used. Univariate and multivariate regressions were also performed in order to investigate the effect of patients’ characteristics and of technical differences of each device on their proper use.

**Results:**

Three hundred thirty-three consecutive out-patients (age range 55–58 years, and well matched for gender), with persistent airway limitation of different severity were investigated, suffering from bronchial asthma (*n* = 175) or from chronic obstructive pulmonary disease (COPD) (*n* = 158). In particular, 127 patients (38 %) tested the three DPIs, while 110 (33 %) tested Breezhaler and Genuair, and 96 (29 %) Breezhaler and Handihaler. More than 50 % of patients who tested all devices preferred the Genuair and perceived this device as the easiest to use. The nurse’s judgement confirmed their opinion. When compared to the other two DPIs, Genuair proved the least problematic either according to the patients’ judgement and to the nurse’s opinion. Mean number of attempts aimed to achieving the first proper actuation was lower with Genuair than with Breezhaler and Handihaler (1.5 vs 2.5–2.6, *p* < 0.0001). Finally, Genuair also proved the easiest to use and the least problematic according to the nurse judgement (0.0001), the most easily learned (0.0001), and that one with a successful rate of more than 56 % at the first attempt. Breezhaler and Handihaler needed an average of about one additional attempt to be used properly (*p* < 0.0001), and their usability proved significantly more difficult (OR of successful rate between 0.15 and 0.17, *p* < 0.001). In general, older patients needed more attempts to perform their first proper inhalation; their successful rate was lower, and they needed more time to learn how to use devices properly: with Genuair these differences were minimized.

**Conclusions:**

The possibility of grading objectively the performance of different DPIs in terms of their usability and therapeutic convenience in daily life represents a crucial operational opportunity to pursue. To note that a substantial discrepancy exists between the patients’ belief “at glance” and the patients’ effective usability with can be registered with some devices. From a general point of view, devices requiring less manual actions for their actuation confirmed their better usability and proper handling after less attempts. In particular, Genuair came out as the most preferred DPI also when several different aspects of preference and usability are assessed objectively and compared.

## Background

Respiratory drugs are preferably delivered via the inhalation route both in bronchial asthma or in chronic obstructive pulmonary disease (COPD) because they target directly the lungs; offer a more rapid onset of action; allow the use of smaller doses, and consent a better efficacy-to-safety ratio compared to systemic treatments [1–4].

The inhalation devices represent *per sé* critical factors because they are able to affect the therapeutic outcomes substantially and independently of the drug used. Actually, several aspects specifically related to the devices can contribute to the effectiveness of treatment, such as their capability to consent the inhalation of a sufficient respirable fraction of the drug (with a particle size ≤ 6 μ); the dose reproducibility; the dose precision and stability, and the comfortable usability in daily life use, particularly in elder patients [5–8].

The development of the Dry Powder Inhalers (DPIs) represented a substantial forward step in the evolution of the inhalation strategy: they do not contain propellants; they optimize the consistency of inhaled drug and the extent of its lung deposition, and generally minimize the role of patient’s cooperation and comprehension in limiting the effectiveness of inhalation [9, 10].

The patients’ usability and preference (PUP) for inhaled devices in general, and for DPIs in particular, still represent two relevant issues to assess as their role still is high indeed and can influence the extent of the patient’s adherence to inhalation treatment and, consequently, the therapeutic outcomes [1, 11–13].

The PUP level is usually assessed by means of validated tools (such as, questionnaires) [14, 15]. The *Handling Questionnaire* is a validated questionnaire which was specifically designed to identify and compare the determinants of choice and acceptability of different inhalation devices in patients with persistent airflow limitation, namely bronchial asthma and chronic obstructive pulmonary disease (COPD) [14, 15].

Aim of the study was to assess by means of the *Handling Questionnaire* and compare the PUP of three different DPIs in out-patients with persistent airflow limitation due to asthma or COPD, according to a controlled design where the patient’s opinion had to be compared to the objective judgment of a supervising expert nurse.

## Methods

In order to investigate the patients’ PUP of different DPIs, the *Handling Questionnaire* was administered (from September to December 2015) to consecutive patients, with persistent airway obstruction needing an inhalation therapy*.* The *Handling Questionnaire* is anonymous; it allows the assessment of different domains of PUP, and takes also into account the patients’ age and gender, together with their previous experience with and their previous educational approach to inhalation devices. Patients with and without previous experience and/or instruction to inhalation devices were included in order to investigate the duration of their instruction.

PUP was measured for three different DPIs: the Breezhaler, the Genuair, and the Handihaler. These devices were chosen because they need a different number of actions for their actuation (such as: 7; 3, and 8, respectively), and are also differently characterized in terms of their intrinsic resistance (such as: very low (0.017 kPa^0.5^ L/min); medium (0.031 kPa^0.5^ L/min), and high (0.058 kPa^0.5^ L/min, respectively).

The study plan consisted of three steps. In the first step, the functioning of each device was shown to each patient in random order by a professional nurse, highly expert in educational programs and specifically trained in the technical and the psychological aspects of the study. Patients were then required to report their preference simply on the basis of their opinion “at glance”, together to the reason of their preference. In the following phase of the study (that is, after the nurse had instructed each patient on the functioning of each device), each patient had to prepare the actuation from each device by him/herself, while the nurse was monitoring his/her technicality. The nurse had also to note the critical issues; to count the number of attempts needed for actuating the device properly, and to measure the time (in sec.) required for these procedures. During this step of the study (that is once experienced the device directly), each subject was required to report once again his/her preference and to specify the reason, but on the basis of a personal, practical experience. At the end of this step, the nurse added her comments to those of each patient, in order to compare the two opinions at the end of the study. In the final step, patients were required to give for the third time their preference, concerning different aspects of acceptance and usability of each device.

As the three devices to compare were different in terms of procedures and number of actions for their actuation, the time spent in explaining the correct functioning of each device and in instructing each patient to the use of each device was measured (in sec.) and compared, together with the time required to each patient for actuating each device effectively. Data were collected up to three attempts per device.

### Statistics

Descriptive statistics are presented as mean ± standard deviation (SD), or simple percentage, as appropriate. Possible differences among devices were analyzed using the Welch test, the Wilcoxon test or the ANOVA test as appropriate. The *χ*^2^ test was used for categorical variables. A *p* <0.05 was accepted as the level of statistical significance for all tests. All analyses were performed using computer software R 3.1.2 [16]. Univariate and multivariate regression were also performed in order to investigate the effect of patients’ characteristics and of technical differences of each device on their proper use. Factors associated with the proper use of DPIs were analyzed using univariate and multivariate linear regressions. Variables with *p* less than 0.1 in the univariate analysis were included in the multivariate model from which we extracted only statistically significant values (*p* < 0.05) by using the stepwise selection technique (backward).

## Results

The overall patients’ sample consisted of 333 consecutive out-patients with persistent airflow limitation of different severity, either due to bronchial asthma (*n* = 175), or to chronic obstructive pulmonary disease (COPD) (*n* = 158). In particular, 127 patients (38 %) tested all the three DPIs, while 110 (33 %) tested Breezhaler and Genuair, and 96 (29 %) Breezhaler and Handihaler.

The characteristics of the patients’ sample and data of patients’ usability for each device are reported in Table 1, together with the mean duration of the three nurse’s educational explanations and of the corresponding number of patients’ operational attempts. To note that all the patients recruited had the opportunity to use all these DPIs in their past, but also other DPIs (namely, the Diskus and the Turbohaler), at a variable regimen and duration, resulting the corresponding distribution randomly comparable within the three sub-groups tested in the present study.

**Table 1:** Characteristics of tested devices, baseline characteristics of patients, and differences in usability

		Breezhaler	Genuair	Handihaler	*p*
Device characteristics					
Manoeuvres (n)^a^		7	3	4	
Time spent by nurse with patients during training^b^ (min)	1^st^ attempt	2.8 ± 0.1	1.3 ± 0.1	1.4 ± 0.1	*p* < 0.0001
	2^nd^ attempt	4.2 ± 0.2	1.8 ± 0.1	2 ± 0.2	*p* < 0.0001
	3^rd^ attempt	5.0 ± 0.3	2.1 ± 0.2	2.7 ± 0.2	*p* < 0.0001
Patient characteristics					
Patients (N)		333	237	223	
Age (years)		55.2 ± 18.3	54.8 ± 17.5	57.8 ± 17.9	NS
Sex (% male)		46.5 %	49.4 %	43.9 %	NS
Disease (% COPD)		47.4 %	47.7 %	51.6 %	NS
Previous experience with DPI		63.7 %	66.7 %	64.6 %	NS
Previous instruction to use of DPI		60.7 %	64.1 %	62.8 %	NS
Usability					
N. attempts before achieving the first proper inhalation		2.6 ± 1.1	1.6 ± 0.8	2.5 ± 1.1	*p* < 0.0001
Successful rate at the first attempt (%)		18.0 %	55.7 %	19.3 %	*p* < 0.0001
Total time required for the first proper inhalation^b^ (min)		10.3 ± 5.0	2.5 ± 1.6	4.9 ± 2.7	*p* < 0.0001

*DPI* dry powder inhaler

^a^Number of operations to prepare the inhalation

^b^Including patient time and nurse demonstration

**Fig. 1:**
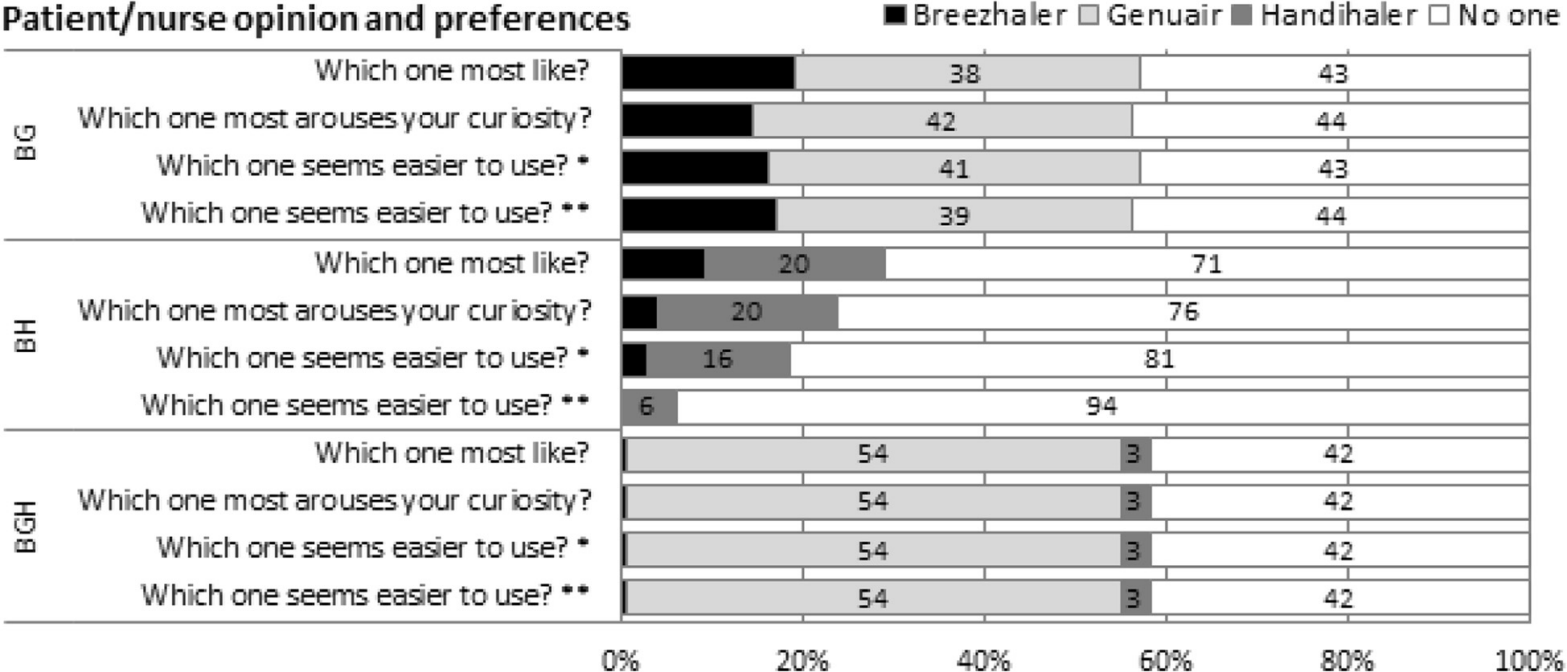
Patient/nurse opinion and preferences. *patient judgement (after the nurse instruction), ** nurse report. BGH: patients tested all devices, BG: patients comparing Breezhaler with Genuair, BH: BG: patients comparing Breezhaler with Handihaler

At baseline, patients in the three device sub-groups were well matched in terms of mean age (ranging 55–58 years); gender; original cause for their airway obstruction; previous experience with and instruction to DPIs (all *p* = ns) (Table 1). More than 50 % of patients who tested all devices preferred the Genuair and perceived this device as the easiest to use, the nurse’s judgement confirming their opinion (Fig. 1, where the comparison among Breezhaler, Genuair, and Handihaler is reported). The remaining patients appear hesitant in judging devices and less than 5 % chose Breezhaler or Handihaler. When only Breezhaler and Genuair were tested, the result was similar (Fig. 1, see comparison Breezhaler vs Genuair), while, if Genuair was not included in the comparison (Fig. 1, see comparison Breezhaler vs Handihaler), about 80 % preferred to not choose any device.

**Fig. 2:**
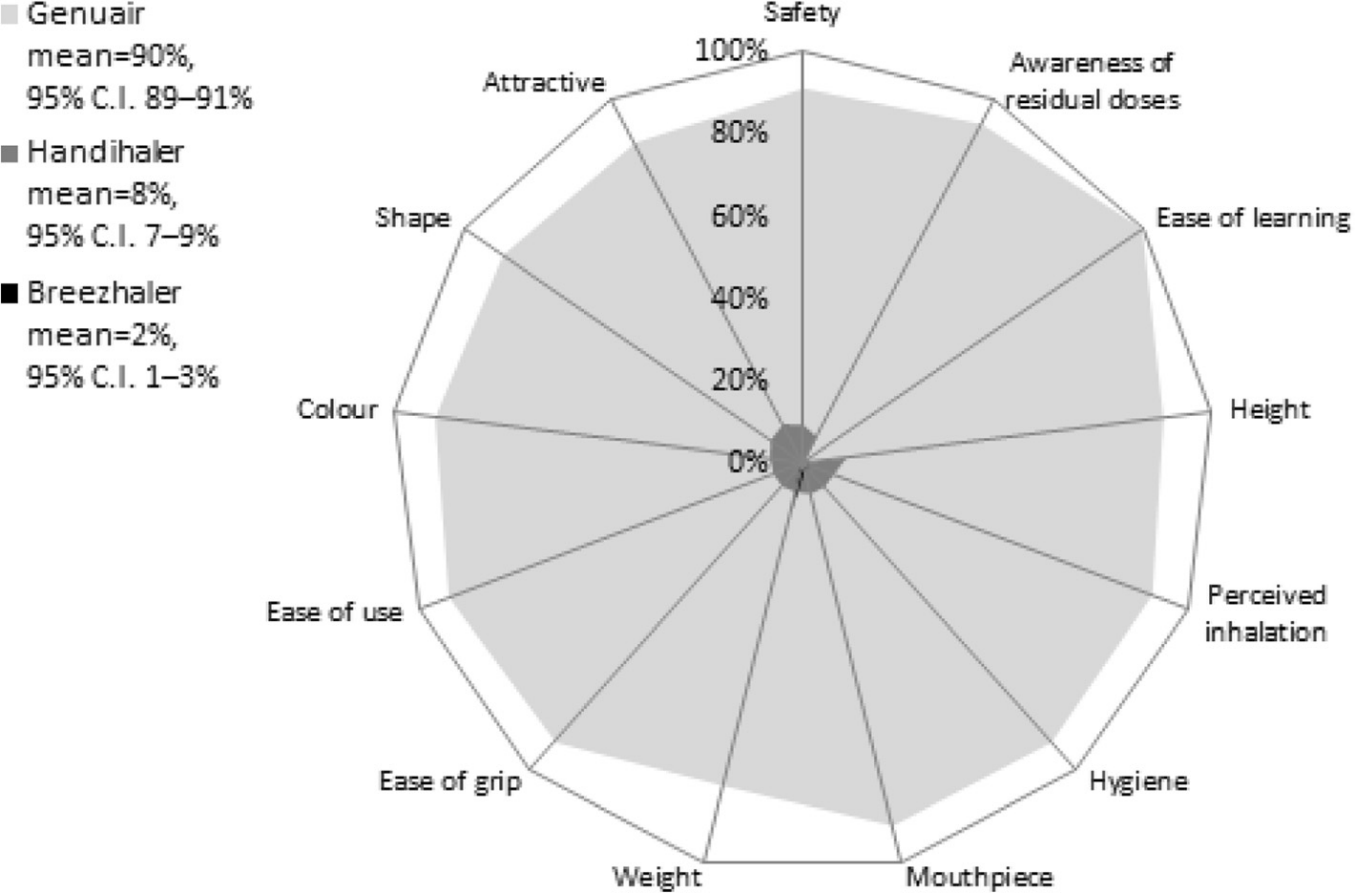
Response to the question “Which device offers the most convenience in terms of…?”: the patients’ opinion

Genuair was the most preferred in terms of appearance, comfort, safety and convenience (Fig. 2); about 90 % of patients chose Genuair on the basis of the series of preference questions “Which device offers the most convenience in terms of…?” followed by Handihaler (8 %) and Breezhaler (2 %) (Fig. 2).

**Fig. 3:**
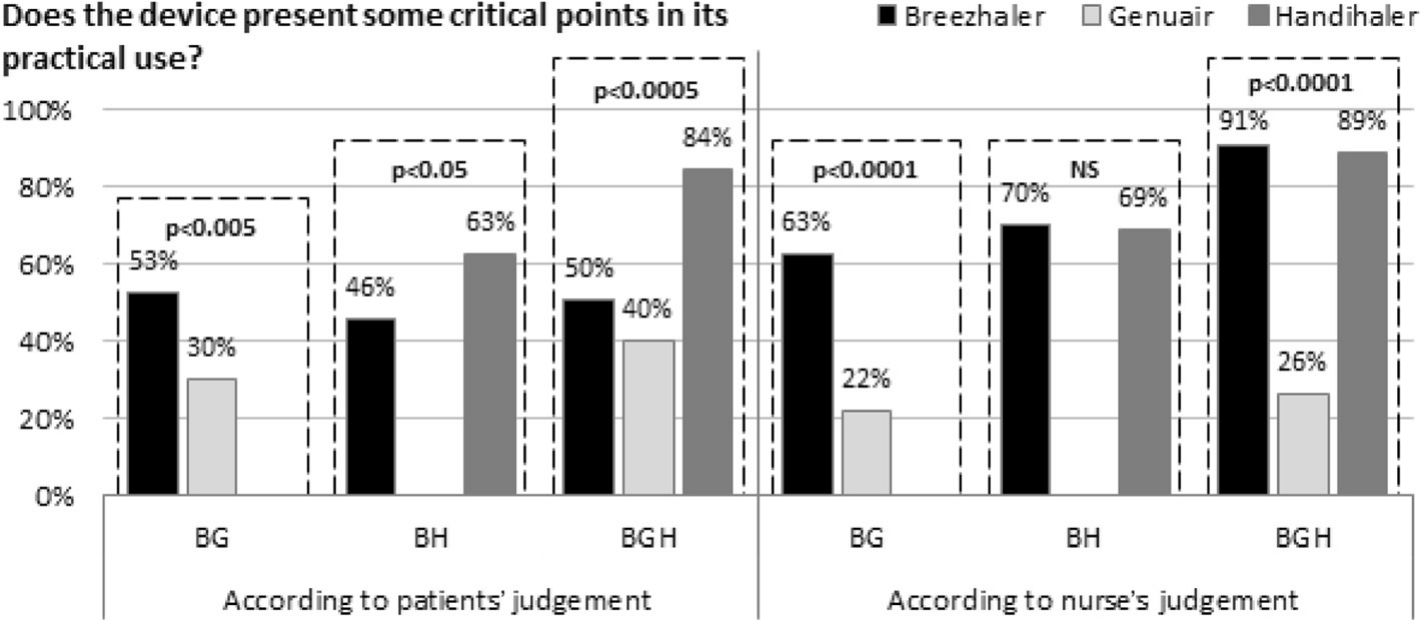
Presence of any problems found after the use of devices: the patients’ vs the nurse’s judgement. BGH: patients tested all devices, BG: patients comparing Breezhaler with Genuair, BH: BG: patients comparing Breezhaler with Handihaler

When compared to the other two DPIs, Genuair proved the least problematic either according to the patients’ judgement and to the nurse’s opinion (Fig. 3). In general, patients seemed to underestimate the difficulties encountered when practicing the Breezhaler: actually, approximately 50 % of patients who tested Breezhaler found some substantial difficulties (both considering all patients and the subgroup analysis), while this proportion increased up to 90 % according to the nurse’s judgement (Fig. 3). There was a perfect agreement between patients and nurse in judging the use of Handihaler as quite difficult.

The number of attempts required to patients for preparing the first proper inhalation represents a very important indicator of efficiency and practicality of the devices investigated in the present study. Mean number of attempts before achieving the first proper actuation was lower with Genuair than with Breezhaler and Handihaler (1.5 vs 2.5–2.6, *p* < 0.0001). Furthermore, 56 % of patients learned how to use Genuair properly after the first demonstration, while more than 80 % of patients were unable to use the other two devices properly after the first demonstration (Table 1). The time for the nurse’s explanation and that needed to patients for preparing the inhalation with Genuair and Handihaler proved similar. Nevertheless, the total time spent in learning how to use the former device properly was significantly lower than that of the latter device (*p* < 0.0001). The corresponding total time for Breezhaler was the highest (more than 10 min), due to the highest number of manoeuvres required, as well as to the longest explanation (Table 1).

**Fig. 4:**
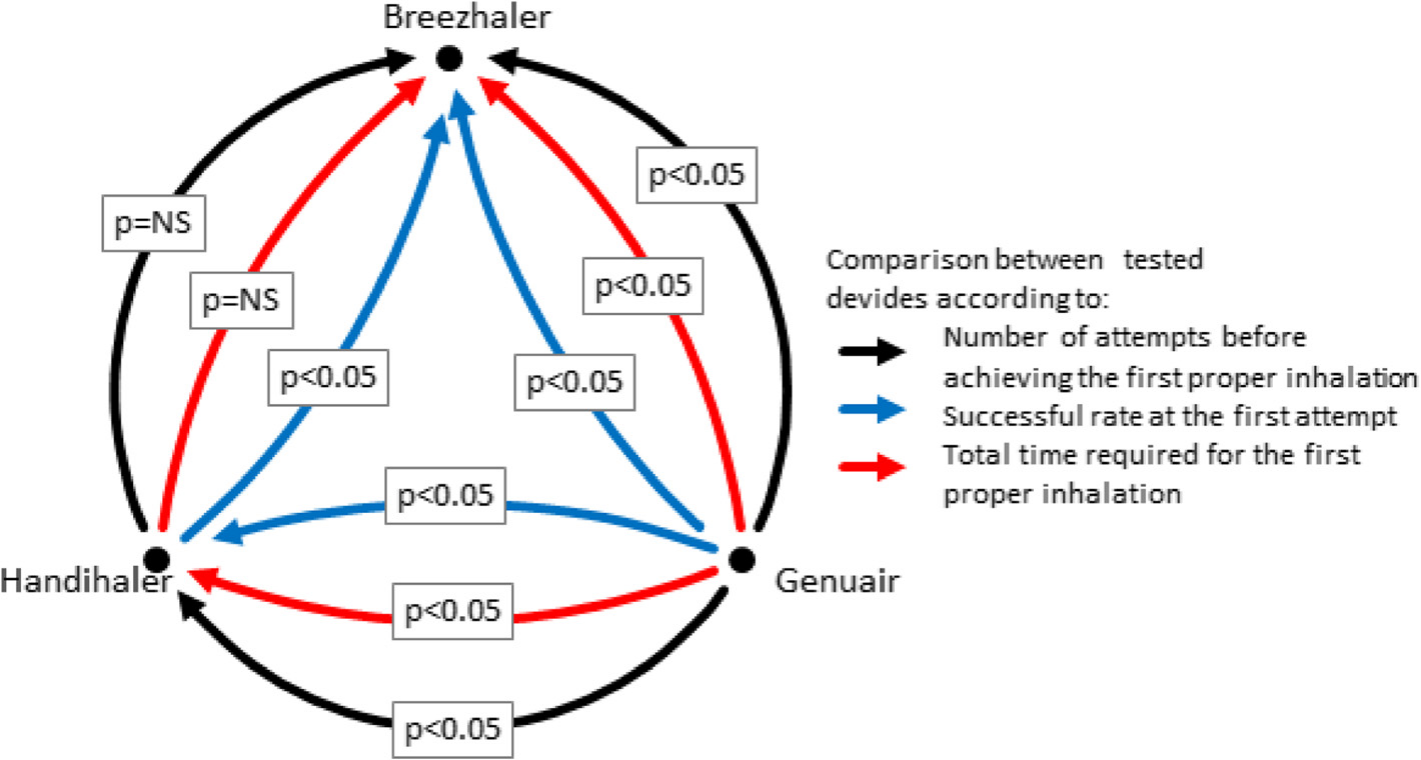
Pairwise comparison between the three devices investigated: arrows’ direction represents which device is superior (with statistical significance according to *p*); e.g. the black arrow joining Genuair and Breezhaler means that Genuair results statistically superior (*p* < 0.05) to Breezhaler according to the number of attempts before achieving the first proper inhalation (such as, Genuair needs less attempts)

Univariate and multivariate analyses showed that some significant differences in device usability were mainly due to patients’ age and to devices themselves (Table 2). Older patients needed more attempts to perform the first proper inhalation (0.07 every 5-years increment, *p* < 0.0001). Moreover, their successful rate was lower (OR = 0.84, *p* < 0.0001) and they needed more time to learn how to use devices properly (an average of 0.25 min, *p* < 0.0001). Breezhaler and Handihaler needed an average of about one additional attempt to be used properly (*p* < 0.0001), and their usability proved significantly more difficult (OR of successful rate between 0.15 and 0.17, *p* < 0.001). On the contrary, a previous experience in the use of DPIs did not seem to facilitate their proper use (OR = 0.64, *p* < 0.0001). The overall comparison among the three devices is reported in Fig. 4: Genuair resulted the easiest to use and the fastest to learn, while no significant difference was detected between Breezhaler and Handihaler either in the number of attempts needed to actuate the first proper inhalation or in the successful rate at the first attempt. In particular, as Breezhaler required the longest to explain and to use, the total time needed for the first proper inhalation resulted significantly lower with Handihaler than with Breezhaler (*p* < 0.0001).

**Table 2:** Predictors of proper inhalation achieving: results of univariate and multivariate linear regressions on patients characteristics and tested devices

Attempts before achieving the first proper inhalation (N)	Univariate analysis		Multivariate analysis	
	delta (95 % C.I.)	*p*	delta (95 % C.I.)	*p*
Age ^a^	0.08 (0.05─0.10)	< 0.0001	0.07 (0.05─0.09)	< 0.0001
Sex (female)	−0.03 (-0.18─0.12)	NS		
Disease (COPD vs asthma)	0.36 (0.21─0.51)	< 0.0001		
Previous experience with DPI	0.14 (-0.02─0.30)	NS		
Previous instructed to DPI	0.12 (-0.04─0.28)	NS		
Device (Breezhaler vs Genuair)	1.01 (0.84─1.18)	< 0.0001	1.00 (0.84─1.16)	< 0.0001
Device (Handihaler vs Genuair)	0.91 (0.73─1.10)	< 0.0001	0.87 (0.69─1.05)	< 0.0001
Successful rate at the first attempt (%)	Univariate analysis		Multivariate analysis	
	OR (95 % C.I.)	*p*	OR (95 % C.I.)	*p*
Age ^a^	0.85 (0.81─0.89)	< 0.0001	0.84 (0.80─0.88)	< 0.0001
Sex (female)	0.96 (0.71─1.3)	NS		
Disease (COPD vs asthma)	0.43 (0.31─0.58)	<0.0001		
Previous experience with DPI	0.59 (0.43─0.81)	< 0.001	0.64 (0.45─0.92)	< 0.05
Previous instructed to DPI	0.64 (0.47─0.88)	< 0.01		
Device (Breezhaler vs Genuair)	0.17 (0.12─0.25)	< 0.0001	0.15 (0.10─0.22)	< 0.0001
Device (Handihaler vs Genuair)	0.19 (0.12─0.29)	< 0.0001	0.17 (0.11─0.27)	< 0.0001
Total time required for the first proper inhalation^b^ (min)	Univariate analysis		Multivariate analysis	
	delta (95 % C.I.)	*p*	delta (95 % C.I.)	*p*
Age ^a^	0.24 (0.14─0.33)	< 0.0001	0.25 (0.18─0.32)	< 0.0001
Sex (female)	−0.29 (-0.99─0.40)	NS		
Disease (COPD vs asthma)	1.12 (0.42─1.81)	<0.005		
Previous experience with DPI	0.26 (-0.47─0.99)	NS		
Previous instructed to DPI	0.17 (-0.55─0.88)	NS		
Device (Breezhaler vs Genuair)	7.76 (7.15─8.37)	< 0.0001	7.74 (7.15─8.33)	< 0.0001
Device (Handihaler vs Genuair)	2.45 (1.78─3.12)	< 0.0001	2.30 (1.65─2.95)	< 0.0001

*DPI* dry powder inhaler, *NS* not significant

^a^Every 5-years increment

^b^including patient time and nurse demonstration

### Subgroup analysis: asthma vs COPD patients

Patients with asthma were significantly younger than COPD patients (44 ± 16 vs 68 ± 11, *p* < 0.0001) and less trained in the use of DPIs: less previous experienced (56 vs 72 %, *p* < 0.005), and less instructed in their use (55 vs 67 %, *p* < 0.05). No differences were be observed by sex (Table 2).

Despite the difference in asthma and COPD patients, in both groups the Genuair resulted the most preferred and teh Handihaler the most dificult to use.

In general terms, asthma patients had less difficulties in learning how to use the devices properly: the mean number of attempts was lower in asthma than in the COPD group (2.1 ± 1.1 vs 2.5 ± 1.1, *p* < 0.0001). Correspondingly, the successful rate at the first attempt was also higher (38 vs 21 %, *p* < 0.0001).

According to these results, the time spent by each patient to learn how to use devices properly was lower in the asthma group by an average of more than 1 min (5.9 ± 4.8 vs 7.0 ± 5.1 min, *p* < 0.001).

## Discussion

Even if respiratory drugs should be preferably delivered via the inhalation route, the choice of the inhaler device to prescribe frequently is empirically guided in real life, and the determinant of choice are usually completely independent of the knowledge of the technological characteristics of devices and of their performance [17–20].

It is well known since long ago that specific education is essential in order to maintain and improve the patients' usability of prescribed device(s), even if it represents a relevant issue in usual daily activity because it is time consuming [21], and specific professional figures are frequently missing. Furthermore, it should be considered that also health care professionals (such as: GPs, medical students, respiratory physiotherapists, pharmacists, and even nurses and lung physicians) frequently prove an inadequate skill in using inhalation devices [13], and then they cannot support their patients who are prescribed these devices for any aid.

The determinants of patients’ choice and preference represent a crucial issue which can affect the outcomes of the therapeutic strategy, particularly if the device requires a complicated sequence of manoeuvres for its proper actuation, and the procedures are not preceded or associated to a sufficient patient’s instruction [4, 22].

As patients with persistent airflow limitation, either asthma or COPD patients, usually need long-term inhalation treatments and should then be familial with their device(s) for long periods, their PUP represent a crucial point indeed in terms of their therapeutic strategy. Unfortunately, the patient’s opinion was only episodically regarded as a crucial variable which can influence the effectiveness of their treatment, even substantially [17–20]. This aspect is of greater value when considering that, at present, the majority of molecules available on the market are only delivered through a fixed branded device, which cannot be changed at pleasure, and the possible combination of a particular molecule with a particular device is not possible in the great majority of cases yet.

The interest in patient’s preference revamped in recent years [21, 23–27] due to the increased awareness that PUP would contribute to a better adherence to the therapeutic strategy, thus leading to increased effectiveness of treatment.

Just stemming from this evidence, the devices’ usability and the understanding of procedures for their proper actuation are of great value because they can greatly contribute to differentiate the performances of different DPIs. The possibility of grading objectively the performance of each device in terms of both usability and therapeutic convenience in daily life would represent a strong operational index [22, 28], particularly when derived from specific, comprehensive, and validated investigational instruments [14, 15, 21, 23].

Results of the present study point out to the real differences assessed by the *Handling Questionnaire* among the devices when compared in terms of patient’s preference and usability. Genuair was the most preferred, and the device which was characterized by the lowest degree of difficulties in understanding the manoeuvres needed for actuating the inhalation properly and effectively. Moreover, Genuair also proved the easiest to use and the least problematic according to both the patients’ and the nurse’s judgement (0.0001), the most easily learned (0.0001), and that one with a successful rate of more than 56 % at the first attempt. These data are confirming results of some previous studies which documented Genuair as the most preferred DPI when compared to Breezhaler and Handihaler [25–27, 29].

In terms of preference, to note that sometimes there is a substantial discrepancy between the patients’ belief “at glance” and the patients’ effective usability with some devices in real life. It is the particular case of Breezhaler which confirms again the existence of conflicting data when the patients’ opinion “at glance” is compared to the objective opinion of the nurse who is carefully attending all the steps of the patients’ procedures with each device tested in the study. This particular issue is a true crucial point because, if the effectiveness of inhalation treatments is highly depending on the proper use of devices (such as, of DPIs in the case), both the patients’ and the health care professionals’ judgment of preference is frequently based on simplistic criteria which are mainly founded on subjective perceptions rather than on effective handling skills.

On the other hand, both the duration of the nurse’s explanation and the number of actions required for the actuation of devices proved statistically related to the number of patients’ attempts for the first proper actuation and also to the probability of a successful actuation at the first patient’s attempt.

Data of the present investigation confirmed that the patient’s criteria of preference and usability are not influenced substantially by a previous generic experience with DPIs. Actually, only the successful rate at the first attempt seems significantly affected according to the univariate and multivariate regressions, while the number of attempts for the first proper inhalation and the total time required for the first proper inhalation resulted not significantly changed. On the other hand, no data were available concerning the duration and the quality of the instruction previously received by patients included in the study. This evidence tends to strongly emphasize how volatile can be the effects of the educational approach to inhalation devices in real life when not institutionally structured or carried out by non-professionals figures. Obviously, the outcomes of any inhalation treatment can be affected particularly in those patients suffering from persistent airway flow limitation who need long-term therapeutic strategies.

From a general point of view, devices requiring less manual actions for their actuation confirmed their better usability and proper handling after less attempts. In particular, Genuair came out as the most preferred DPI also when several different domains of preference were investigated and compared, such as from aesthetic attractiveness up to the perception of inhalation and the mouthpiece comfort.

Subgroup analyses showed slight differences between asthma and COPD patients which are likely due to the fact that asthma subjects were significantly younger. Actually, asthma group proved to have less difficulty in learning how to use devices and they prove a higher successful rate at the first attempt than COPD patients. While COPD patients perceived the ease of use for B and C device equally, asthma patients preferred device B significantly.

The preference for Genuair when compared to other DPIs was already assessed in generic terms for COPD patients [23], but the patient’s point of view was never compared with that of an expert nurse previously. Furthermore, in the present study the time spent for instruction and the number of attempts for achieving the proper actuation were investigated analytically by linear and logistic regression in order to define the possible influence of the patient’s preference and acceptability. The comparison of Genuair vs Breezhaler and Handihaler was never carried out previously at our knowledge, even if Breezhaler was proved much less preferred than Genuair in a recent study aimed to assess the patient’s satisfaction and the inhaler technique errors in COPD with these two devices [29].

## Conclusions

DPIs, even though belonging to the same family of inhalation devices, are characterized by several technological differences which make each device different from the others of the family. Their gripping, size, manoeuvres for actuation, understanding of inhalation procedures represent some relevant characteristics which can change substantially from each other, and then the patients’ acceptability and usability can change accordingly.

Only objective and measurable indicators should be used for assessing and comparing the real-life performances of DPIs in terms of patients’ preference and usability. In order to provide a comprehensive assessment of each device performance, the number of patients’ attempts for the first proper actuation, together with the successful rate at the first attempt, and the total time required for the first proper actuation represent the three main determinants of usability which should greatly contribute to the objective judgment of any DPI convenience in real life.

When compared to Breezhaler and Handihaler in terms of these aspects, Genuair proved the DPI characterized by the highest level of preference, usability, and convenience in real life by patients with persistent airflow limitation.

## Abbreviations

PUP, patient’s usability and preference; DPIs, dry powder inhalers.
